# Targeting Myeloid Cells in Combination Treatments for Glioma and Other Tumors

**DOI:** 10.3389/fimmu.2019.01715

**Published:** 2019-07-23

**Authors:** Andy S. Ding, Denis Routkevitch, Christina Jackson, Michael Lim

**Affiliations:** Department of Neurosurgery, Johns Hopkins School of Medicine, Baltimore, MD, United States

**Keywords:** combination immunotherapy, myeloid therapy, glioma, myeloid-derived suppressor cells, checkpoint inhibitors, radiation, chemotherapy, tumor-associated macrophages

## Abstract

Myeloid cells constitute a significant part of the immune system in the context of cancer, exhibiting both immunostimulatory effects, through their role as antigen presenting cells, and immunosuppressive effects, through their polarization to myeloid-derived suppressor cells (MDSCs) and tumor-associated macrophages. While they are rarely sufficient to generate potent anti-tumor effects on their own, myeloid cells have the ability to interact with a variety of immune populations to aid in mounting an appropriate anti-tumor immune response. Therefore, myeloid therapies have gained momentum as a potential adjunct to current therapies such as immune checkpoint inhibitors (ICIs), dendritic cell vaccines, oncolytic viruses, and traditional chemoradiation to enhance therapeutic response. In this review, we outline critical pathways involved in the recruitment of the myeloid population to the tumor microenvironment and in their polarization to immunostimulatory or immunosuppressive phenotypes. We also emphasize existing strategies of modulating myeloid recruitment and polarization to improve anti-tumor immune responses. We then summarize current preclinical and clinical studies that highlight treatment outcomes of combining myeloid targeted therapies with other immune-based and traditional therapies. Despite promising results from reports of limited clinical trials thus far, there remain challenges in optimally harnessing the myeloid compartment as an adjunct to enhancing anti-tumor immune responses. Further large Phase II and ultimately Phase III clinical trials are needed to elucidate the treatment benefit of combination therapies in the fight against cancer.

## Introduction

The recent rise to prominence of immunotherapy into the forefront of cancer treatment has resulted in an abundance of research aimed at harnessing various components of host immunity in anti-tumor treatments. Immunotherapy efforts have historically focused on boosting the activities of the lymphocyte compartment, specifically CD8^+^ cytotoxic T lymphocytes (CTLs), with the use of immune checkpoint inhibitors (ICIs), chimeric antigen receptor (CAR) T cells, peptide vaccines, and oncolytic viral therapy. While T cell-based therapies, particularly those involved with immune checkpoint inhibition, have shown improved survival and tumor regression in multiple systemic cancers including non-small cell lung cancer and melanoma, their benefits are not universal. The efficacy of T cell-based therapies is predicated on the presence of tumor infiltrating lymphocytes (TILs); tumors with fewer TILs are less responsive to these therapies and are considered immunologically “cold tumors.” Myeloid cells are a significant, yet sometimes overlooked component of immunotherapy. In normal physiologic states, myeloid cells play an important role in innate immunity while also contributing to the adaptive immune response through antigen presentation. However, in the setting of cancer, they can be induced by a multitude of factors to adopt an immunosuppressive phenotype that can lead to the inhibition of anti-tumor responses by CTLs. These suppressive myeloid cells are particularly abundant in immunologically cold tumors prompting increasing efforts to target these cells to improve the efficacy of immunotherapy. Furthermore, there is increasing evidence that adjuvant therapies such as chemotherapy and radiation can have conflicting effects on the efficacy of immunotherapy, with the potential to be synergistic or antagonistic when reshaping the myeloid population. Therefore, it is critical to understand the interplay between the tumor, immune cells, and adjuvant therapy to fully optimize the efficacy of immunotherapy.

The myeloid compartment is especially relevant in the study of gliomas, including glioblastoma (GBM), which is the most aggressive and most common primary central nervous system (CNS) malignancy in adults with a dismal median overall survival of 12–15 months even with the current standard care of surgery followed by adjuvant chemoradiation (1). A growing body of evidence has highlighted the poor immunogenicity of GBM with a paucity of CD8^+^ CTLs, relative abundance of Foxp3^+^ regulatory T cells (Tregs), high infiltration of tumor-associated immunosuppressive macrophages and microglia (TAMs), and presence of myeloid-derived suppressor cells (MDSCs) (2–5). These factors are likely responsible for the minimal efficacy of T cell-based therapies in GBM. MDSCs are divided into two groups: granulocytic/polymorphonuclear (PMN-MDSCs) and monocytic (M-MDSCs) which are phenotypically and morphologically similar to neutrophils and monocytes, respectively. Although similar to typical myeloid cells, M-MDSCs are distinguishable from monocytes by low/absent expression of HLA-DR, while PMN-MDSCs are distinguishable from neutrophils by LOX-1 expression (6). Studies have shown TAMs and MDSCs to constitute a large proportion of tumor infiltrating immune cells in the GBM tumor microenvironment (TME) (7), ranging from 30 to 90% in human GBM samples, with CD11b^+^ MDSCs comprising the majority of infiltrating inflammatory cells in human gliomas (8, 9).

Unique to gliomas and other brain tumors, a significant portion of the tumor-associated myeloid compartment consist of microglia, the resident macrophages of the CNS. Historically, these tumor-associated macrophages and microglia have been used interchangeably. The advent of genome-wide microarray and single-cell RNA sequencing analyses have allowed for phenotypic and transcriptomic differentiation between these two populations. These studies have demonstrated that microglia are characterized by low expression of CD45 and major histocompatibility complex II (MHCII), absence of C-C motif chemokine receptor 2 (CCR2), and high expression of purinergic receptor P2RY12, C-X3-C motif chemokine receptor 1 (CX3CR1), and transmembrane protein 119 (TMEM119), while blood-derived macrophages demonstrate high expression of CD45, MHCII, and tyrosine-protein kinase Mer (MERTK) (10–12). Single-cell RNA sequencing of gliomas have also shown that microglial TAMs are enriched in the leading edge of the tumor and surrounding white matter, while blood-derived TAMs are more often found within regions of microvascular proliferation and peri-necrotic regions within the core of the tumor. This is correlated with higher expression of pro-inflammatory factors in the periphery and anti-inflammatory factors in the core (13). In fact, TAMs that originate from the blood and migrate to brain tumors, where they adopt a more tissue-specific phenotype, have been shown to have a distinct metabolism as well as increased expression of immunosuppressive markers when compared to microglia (14). Additionally, as glioma grade increases, the ratio of blood-derived TAMs to microglia concurrently increases (15). However, despite the increased tendency in microglia toward a pro-inflammatory phenotype, both cell types have the potential for tumor-based induction toward MDSCs and can thus be targets for myeloid therapy (16).

For tumors that endorse a myeloid-enriched TME, like gliomas, therapies that are able to re-program the immunosuppressive myeloid population back to immunostimulatory phenotypes or limit the function of TAMs and MDSCs may enhance the effectiveness of and reduce resistance to existing therapies. This review aims to highlight potential targets for myeloid therapy, with a specific focus on recent efforts in combining myeloid targeted therapy with other treatment options to optimize the efficacy of immune-based therapies.

## Preclinical Glioma Models for Myeloid Study

The most commonly used glioma murine models in preclinical studies of myeloid populations and myeloid-based therapies are orthotopic models that are accomplished by intracranial injection of established glioma cell lines such as GL261 and CT2A. However, these models harbor inherent limitations in representing *de novo* tumorigenesis in the host and have variable immunogenic responses due to the necessity of using immunosuppressed or immunodeficient animal hosts for orthotopic implantation (17–20). To address some of these limitations, genetically engineered models that employ overexpression of relevant oncogenic receptors or downstream signaling pathways, such as replication-competent avian sarcoma-leukosis virus (RCAS) engineered with the sleeping beauty (SB) transposon, have been developed and result in *de novo* tumor formation (21–24). These genetically engineered mice (GEMs) have the advantage of having the tumor originate from the host's own cells, as well as the utility of using immunocompetent animals to assess tumor immunogenicity and response to therapy, but are poorly reproducible and are more representative of genetic predispositions to cancer rather than random tumorigenesis by point mutation (25). A combination of the two techniques, in which donor mouse cells are transfected with the RCAS system and implanted into recipient mice, has also been explored (11, 26), which improves the correlation to human gliomagenesis, but is limited in reproducibility.

## Targets for Myeloid Therapy

**Figure 1:**
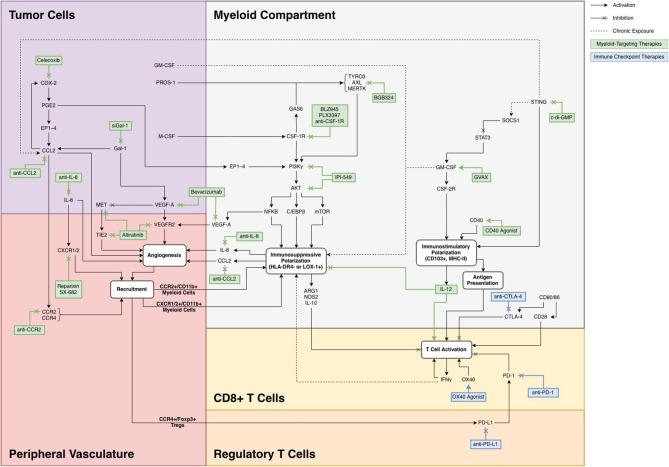
A summary of previously targeted myeloid pathways with potential for combination therapy.

Strategies for targeting the myeloid compartment generally fall into three main categories: (A) modulating the recruitment of MDSCs from peripheral blood; (B) promoting an immunostimulatory phenotype, primarily through maturation of myeloid precursors into inflammatory macrophages and antigen presenting dendritic cells (DCs); and (C) inhibiting the polarization of myeloid cells to MDSCs. The pathways involved in these three methodologies are shown in Figure 1, organized in the context of the TME in which each target is involved.

### Inhibiting the Recruitment of MDSCs

#### CCL2/CCR2

C-C motif chemokine ligand 2 (CCL2, MCP1) was first characterized as a cytokine that interacted with its receptor, CCR2, on peripheral blood monocytes to facilitate chemotaxis to active areas of inflammation (27). In a murine K1492 GBM model, Zemp et al. demonstrated that in addition to recruiting peripheral monocytes to sites of infection, inflammation, and other neuropathological conditions, CCR2 also plays a role in recruiting glioma infiltrating monocytes and macrophages to the TME (28). The authors showed that when oncolytic myxoma virus therapy was given to CCR2-null mice, there was impaired monocyte infiltration and clearance of the virus, leading to increased effectiveness of the therapy and increased survival compared to wild-type mice. Concurrently, Lesokhin et al. confirmed in a B16 melanoma-bearing mouse model that chronic secretion of GM-CSF from the tumor led to recruitment of monocytic MDSCs, characterized by CCR2/CD11b co-positivity, which inhibited TIL proliferation and infiltration in the TME (29). The same group found that while CCR2 was not necessary for MDSC activation, knockdown of CCR2 resulted in a 50% reduction in tumor-infiltrating MDSCs. These results were corroborated by Zhu et al. who directly blocked CCL2 with a monoclonal antibody in C57BL/6 mice bearing intracranial either GL261 or U87 glioma cancer cells and found that blockade of CCL2 led to an increase in median survival in both mouse models (30).

Chang et al. further expanded upon the role of the CCL2/CCR2 axis in glioma immune evasion (31). Using a murine GL261 glioma model, they showed that glioma cells are capable of secreting CCL2 to recruit MDSCs to the tumor site, and that tumor-derived CCL2 can further induce TAMs to secrete CCL2 leading to synergistic tumor immune suppression. In addition to recruiting myeloid cells, the group also found that tumor- and TAM-secreted CCL2 can lead to the recruitment of Tregs through CCR4, further dampening the ability of CTLs to exert anti-tumor effect. This positive feedback loop can help explain the efficacy of CCL2 blockade in anti-tumor response as shown by previous groups. In addition to antagonistic anti-CCL2 antibodies, minocycline has also been shown to inhibit the synthesis of CCL2 by TAMs and has the potential to block this immunosuppressive pathway to work synergistically with current glioma therapeutics (32).

Chen et al. confirmed the relevance of CCL2 in glioma patients by querying the TCGA database and showed that high CCL2 expression was associated with worse prognosis and shorter median survival compared to patients who exhibited low expression of CCL2 (11). These findings suggest that the CCL2/CCR2 axis is a primary mechanism by which glioma cells can recruit MDSCs to promote tumor growth and reduce the effectiveness of anti-cancer therapeutics. The aforementioned studies all highlight the importance of the CCR2 axis in recruiting MDSCs to the tumor and suggest the potential for its blockade in combination with other therapies to potentiate an anti-tumor immune response.

#### VEGF-A/MET/TIE2/VEGFR2

The vascular endothelial growth factor receptor 2 (VEGFR2) pathway has been well-known to stimulate angiogenesis during tumor development. Recent work from Huang et al. has shed new light on VEGFR2, not only as a regulator of angiogenesis in response to hypoxia, but also as an inducer of myeloid differentiation to MDSCs and their subsequent recruitment to the TME (33). The group showed that myeloid-derived hematopoietic cells that express higher levels of VEGFR2 were correlated with higher tumor grade, worse prognosis, and higher rates of tumor progression in glioma patients. In murine glioma models, the same group knocked down VEGFR2 in bone marrow-derived macrophages, which resulted in significantly decreased tumor blood perfusion and tumor volume, as well as a relative absence of tumor-associated MDSCs. These results suggest that VEGFR2 in peripheral myeloid cells aids in MDSC polarization and trafficking. Interestingly, while Piao et al. demonstrated that anti-angiogenic therapy via bevacizumab prolonged survival in murine glioma models, the group also observed an increase in MDSC recruitment and increased expression of transforming growth factor beta 1 (TGFβ1), an immunosuppressive cytokine, in the TME post treatment (34). Similar results of TGFβ1 upregulation were observed by Osterberg et al. in GL261-implanted mice with selective VEGF-A knockout in CD11b^+^ myeloid cells (35). Furthermore, the use of anti-angiogenic agents such as bevacizumab and sorafenib in clinical trials has historically been unsuccessful at improving patient survival in glioma (36, 37). These findings suggest that while conventional anti-angiogenic therapy can lead to decreased tumor blood perfusion and MDSC trafficking, it also triggers the upregulation of compensatory or alternative pathways for angiogenesis, MDSC recruitment, and therapy resistance.

To expand on their initial findings, Piao et al. identified an alternative angiogenic pathway involving MET and TIE2 that was upregulated after bevacizumab treatment (38). This pathway acts on the same effectors as VEGF-A and is therefore also a mechanism through which the TME can recruit MDSCs. In GBM stem cell (GSC) xenograft mouse models, the group found that treatment with altiratinib, a MET/TIE2/VEGFR2 inhibitor, in combination with bevacizumab, significantly prolonged survival compared to either monotherapy (38). Altiratinib alone conferred no survival benefit, supporting the argument that the MET pathway is normally suppressed by VEGF-A activity. As VEGF inhibitors like bevacizumab are often considered in cancer treatment, their potential suppressive functions on the immune system through the expression of MET may provide new insight on mechanisms of tumor recurrence and resistance to therapy.

#### IL-8/CXCR1/2

Interleukin-8 (IL-8, CXCL8) is a chemoattractant cytokine that was originally described to attract and activate peripheral neutrophils and myeloid cells to areas of inflammation by acting on G protein-coupled receptors C-X-C motif chemokine receptor 1 and 2 (CXCR1 and CXCR2) (39). Recent studies have also characterized IL-8 as a tumor-secreted agent that promotes an immunosuppressive TME via MDSC and neutrophil recruitment, as well as tumor angiogenesis (40–42). Importantly, since the rodent genome lacks the IL-8 gene, preclinical studies evaluating the role of IL-8 in MDSC recruitment have been difficult to conduct. To address this obstacle, Asfaha et al. developed a transgenic mouse model (IL-8Tg), in which a bacterial artificial chromosome that encodes for the human IL-8 gene and its regulatory elements is spliced into the mouse genome (43). Using this model, the group found that carcinogen challenge with azoxymethane and dextran sodium sulfate, produced more colorectal tumors in IL-8Tg mice compared to wild-type mice. Furthermore, the group showed that injection of recombinant human IL-8 resulted in increased trafficking of MDSCs to the TME. Alfaro et al. confirmed these findings in BALB/c mice harboring HT29 colorectal adenocarcinoma flank tumors (44), demonstrating that IL-8 induced MDSC migration from the spleen in a dose-dependent fashion. The same group further found that treatment with reparixin, a CXCR1 and CXCR2 inhibitor, abrogated the trafficking of MDSCs in immunocompromised HT29 tumor-bearing mice that underwent IL-8 hydrodynamic gene transfer. In human melanoma-xenografted BALB/c mice, Huang et al. have further shown that IL-8 blockade with the monoclonal antibody ABX-IL8 significantly inhibited tumor growth and decreased angiogenesis, which in turn inhibited MDSC migration (45). By analyzing the expression of biomarkers in human glioma conditioned media, Kumar et al. have shown that IL-8 is a predominant chemokine in the glioma TME, suggesting that glioma-secreted IL-8 helps contribute to MDSC trafficking to the tumor site (46). Pre-clinical studies evaluating the impact of IL-8 blockade on survival have yet to be conducted in glioma models.

#### Gal-1

Galectin-1 (Gal-1) is the prototypic member of a family of lectins that bind to β-galactosides. Recently, Gal-1 has been described as an important regulator of immune cell trafficking and T cell fate (47). Work from Verschuere et al. has elucidated a link between Gal-1-mediated recruitment of immune cells via CCL2 and VEGF-A (48). The group showed that mice implanted with Gal-1 knockdown GL261 glioma cells not only had prolonged median survival compared to wild-type GL261-implanted mice, but also had decreased levels of MDSCs. Reverse transcription polymerase chain reaction (RT-PCR) revealed that Gal-1 knockdown abrogated CCL2 and VEGF-A mRNA expression in the tumor, resulting in decreased recruitment of MDSCs to the TME and decreased angiogenesis, respectively. Interestingly, Gal-1-knockdown mice implanted with Gal-1-expressing GL261 tumors showed no treatment advantage over wild-type mice, emphasizing the importance of Gal-1 specifically in the TME. By cancer database transcriptomic analysis and immunohistochemistry-based quantifications of GL261, Chen et al. further confirmed that *LGALS1*, the gene encoding Gal-1, was significantly correlated with CCL2 and VEGF-A mRNA expression in the tumor (49). In BV2-bearing mice cells, the same group also knocked down Gal-1 mRNA expression via RNA interference and observed a resulting decrease in MDSCs in the TME. From these results, the expression of Gal-1 is strongly suggested to be an upstream regulator of CCL2 and VEGF-A expression and subsequent inducer of MDSC and Treg recruitment.

There currently exists a variety of Gal-1 inhibitors, including galactoside-derivatives and peptides (50). Of note, Thijssen et al. treated F9 teratocarcinoma-bearing mice with anginex, a polypeptide angiogenesis inhibitor that binds Gal-1, and showed a 70% decrease in tumor growth compared to control mice (51). Shih et al. observed similar findings with LLS2, a small-molecular inhibitor of Gal-1 that decreased tumor growth in a murine ovarian cancer model (52). Finally, in a GL261 glioma mouse model, Van Woensel et al. have targeted Gal-1 in via intranasal administration of nanoparticles loaded with siRNA against Gal-1 (siGal-1), showing a significant reduction of Gal-1 expression in the TME (53). These recent findings have highlighted Gal-1 as a potential target in limiting tumor growth and recruitment of MDSCs via its downstream effectors.

### Promoting an Immunostimulatory Phenotype

#### GM-CSF

Granulocyte macrophage colony stimulating factor (GM-CSF, CSF-2) has a complex role in the regulation of myeloid cells. On one hand, it is commonly used as a method to increase myeloid cell activation and differentiation into DCs (54–63). On the other, it has been shown to promote myeloid immunosuppression through expression of associated markers and inhibition of T cell activation (24, 64, 65). As such, it is important to consider the context of GM-CSF treatment in order to effectively promote immune stimulation.

A common use of GM-CSF for immune stimulation is through vaccination with irradiated tumor cells that have been genetically modified to express GM-CSF (54–57, 66–68), commonly known as GVAX. This technique is based on the rationale that irradiating tumor cells before vaccination causes effective uptake of tumor antigens by macrophages, granulocytes, and DCs without tumor formation, while the expression of GM-CSF allows for activation of the myeloid and dendritic compartments working synergistically to allow successful antigen presentation to T cells (54, 68). Smith et al. demonstrated increased cytotoxic T cell activity with the administration of either GM-CSF vaccine or interferon gamma (IFNγ) in GL261 murine glioma models (55). Interestingly, the administration of GM-CSF alone also showed increase in Tregs and MDSCs. Combination therapy with GM-CSF and IFNγ showed synergistic effects with significantly prolonged survival and long-term immunologic memory at rechallenge. In this case, it is likely that GM-CSF tumor vaccine alone helped enhance antigen presentation by myeloid cells but was not enough to fully activate T cells against the tumor. Combination therapy with other adjuncts is needed to fully harness the immunostimulatory effects of GM-CSF.

As hinted by Smith's study, GM-CSF can also result in immunosuppression with recruitment of Tregs and MDSCs in other contexts. Sielska et al. showed that mice implanted with GL261 tumors knocked down for GM-CSF had significantly improved survival and decreased MDSC infiltration to the TME (64). However, they also found that GM-CSF secreted from the tumor cells resulted in higher expression of immunosuppressive genes, such as arginase 1 (*ARG1)*, within the myeloid population. Notably, this occurred at later timepoints, indicating that chronic GM-CSF exposure likely led to myeloid immunosuppression. Kohanbash et al. demonstrated that interleukin-4 (IL-4) and IL-4 receptor alpha (IL-4Rα) are likely responsible for this immunosuppressive effect (24). They showed that GM-CSF is expressed in glioma tissues and can induce IL-4Rα expression *in vitro*. Knockdown of IL-4Rα in BALB/c mice with *de novo* SB transposon-induced gliomas subsequently resulted in downregulation of immunosuppressive pathways involving TGFβ, ARG1, and cyclooxygenase-2 (COX-2). Additionally, Ribechini et al. showed that GM-CSF induces MDSC polarization *in vitro* through simultaneous activation of the protein kinase B (AKT) cascade and the interferon regulatory factor-1 (IRF-1) pathway (65). As GM-CSF has been implicated in immunosuppression, it could be useful to combine GM-CSF treatment with IL-4α or AKT inhibitors to minimize pro-tumor effects.

The opposing immunomodulatory effects of GM-CSF are important to consider when administering this therapy. GM-CSF is associated with immunosuppression when secreted by an active tumor, where there are a host of other suppressive factors, and when chronically secreted through activation of pathways such as PI3K/AKT (65) and via expansion of MDSCs (55). When in the context of irradiated tumor cells without inhibitory signals, as in GVAX, however, GM-CSF causes immunostimulation by instigating the expansion of a subset of antigen presenting, activated myeloid cells (68). By providing GM-CSF in a context that maximizes the immunostimulatory effects, it can be possible to improve its efficacy alone as well as in combination with other treatments.

#### STING

Stimulator of IFN genes (STING) is another component in the myeloid compartment that has the ability to both inhibit and stimulate the immune system. STING is activated in the presence of cytosolic DNA, resulting in expression of type I IFNs, with cyclic dinucleotides often used as STING agonists. Ohkuri et al. demonstrated the immunostimulatory effects of STING in SB-induced gliomas by showing that STING knockout resulted in increased infiltration of MDSCs and Tregs and lower infiltration of CTLs. Treatment with the STING agonist cyclic diguanylate (c-di-GMP) resulted in enhanced T cell activity (22). Zhang et al. demonstrated another immunostimulatory function of STING in a nasopharyngeal carcinoma model whereby it inhibited the phosphorylation of signal transducer and activator of transcription 3 (STAT3) in both tumor and myeloid cells through suppressor of cytokine signaling 1 (SOCS1), an intracellular STAT inhibitor (69). This decreased the production of GM-CSF and inhibited the polarization of MDSCs. Foote et al. also demonstrated that STING agonists can promote immunostimulation through increased expression of type I IFNs and increased DC activation (70). However, the group also showed the potential suppressive effects of STING agonists through an increase in myeloid expression of programmed death-ligand 1 (PD-L1), the ligand to programmed cell death protein 1 (PD-1) and an often-targeted immune checkpoint, suggesting the presence of alternative pathways and a potential target for combination therapy. The dual nature of STING was also emphasized by Liang et al. who used an MC38 colon cancer model to show that STING is implicated in myeloid-mediated radioresistance through MDSC recruitment from CCR2 signaling (71).

These results suggest that STING agonist treatment is generally immunostimulatory but can also activate immunosuppressive pathways and interfere with other types of treatment. To optimize the anti-tumor function of this pathway, an agonist could be used in combination with blockade of the suppressive downstream pathways of STING, such as PD-L1 and CCR2. The resulting increase in immune activation could then be used synergistically with other treatment therapies such as ICIs and radiation.

#### CD40

CD40 is a costimulatory protein found on myeloid cells and DCs. Activation of CD40 with its ligand, CD154, or an agonist antibody promotes antigen presentation in these cells (72, 73). As a result, agonistic CD40 antibodies have been explored as an option to decrease immunosuppression and increase T cell activation. Chonan et al. used an anti-CD40 agonist in in several glioma models and showed a modest improvement in survival as a result (74). Shoji et al. used convection-enhanced delivery of anti-CD40 agonist to the tumor site and showed moderately improved survival (75). Both groups showed increased T cell infiltration with CD40 stimulation, but neither reported long-term survivors, indicating that a CD40 agonist on its own is not enough to sustain a full anti-tumor immune response. Kosaka et al. additionally demonstrated that CD40, as part of a combination treatment with COX-2 inhibition, polarized myeloid cells away from a suppressive phenotype (21). From these findings, we conclude that in a combination treatment, CD40 effectively stimulates antigen presentation by myeloid cells and inhibits myeloid-derived suppression. Although survival is enhanced, the lack of long-term survivors suggests that a full immune response is not mounted. By polarizing the myeloid compartment away from the immunosuppressive phenotype, CD40 agonists could increase the efficacy of other treatments, including T cell-based therapies.

#### IL-12

Interleukin-12 (IL-12) is secreted by macrophages and promotes an anti-tumor immune response through stimulation of T cells and natural killer cells. Though there is also evidence showing that IL-12 also influences the myeloid compartment. On its own, IL-12 treatment leads to strong systemic toxicity in humans (76). However, localized expression via intratumoral viral transduction or delivery of IL-12 in the tumor has shown promise as a treatment both as a monotherapy (77) and in combination with checkpoint blockade (78, 79). In these cases, the treatment effect was most likely due to anti-tumor T cell stimulation. However, Elzey et al. showed that IL-12 also has the ability to inhibit MDSCs in the TME in a murine breast cancer model by decreasing expression of suppressive genes like *ARG1* (80). In glioma, Thaci et al. found that IL-12 treatment increased myeloid DCs in the TME (81). Work from both groups highlighted the role of IL-12 as both an inhibitor of MDSC function and a promoter of DC maturation, rendering it a promising candidate for combination therapy with T cell-based immunotherapy.

### Inhibition of MDSC Formation From Myeloid Precursors

#### M-CSF/CSF-1R

Macrophage colony stimulating factor (M-CSF, CSF-1) has been well-characterized as a growth factor that binds to colony stimulating factor 1 receptor (CSF-1R) on macrophages and monocytes to stimulate survival and proliferation (82). Coniglio et al. found that microglia surrounding the tumor expressed CSF-1R and responded to CSF-1 via invasion into the TME (83). In a murine GL261 glioma model, the group also found that treatment with PLX3397, a CSF-1R inhibitor that can cross the blood brain barrier, significantly decreased the proportion of microglia in the TME. Concurrently, they observed less tumor invasiveness post-treatment, compared to control groups that experienced extensive tumor cell migration into the brain parenchyma. Their findings suggest that CSF-1R mediates myeloid invasion into the TME and aids in promoting a pro-tumoral environment. As a follow-up, Pyonteck et al. used another CSF-1R inhibitor, BLZ945, on a murine model of RCAS-human platelet-derived growth factor subunit B (hPDGF-B) induced gliomas, which resulted in significantly improved long-term survival rate of 64.3% and no detectable lesions in 55.6% of asymptomatic mice (84). Interestingly, they found that CSF-1R inhibition did not decrease the number of TAMs, but rather abrogated MDSC polarization by downregulating immunosuppressive genes, such as mannose receptor C-type 1 (*MRC1*), adrenomedullin (*ADM*), coagulation factor XIII A chain (*F13A1*), and *ARG1*, in the myeloid compartment. Yan et al. from the same group observed similar results with PLX3397 in the same hPDGF-B-driven glioma model, showing reduced expression of immunosuppressive genes (85). Furthermore, the group found that PLX3397 was significantly more successful at inhibiting tumor growth compared to receptor tyrosine kinase (RTK) inhibitors vatalanib and dovitinib. However, in an another RCAS-hPDGF-B inducible glioma model, Quail et al. found that although CSF-1R inhibition resulted in tumor shrinkage, secretion of IGF-1 in TAMs, and upregulation of IGF-1R in tumor cells resulted in activation of the PI3K pathway in glioma cells, stimulating tumor rebound growth, and recurrence (86). Co-treatment with an IGF-1R or PI3K inhibitor significantly prolonged median survival in mice treated with PLX3397. These findings demonstrate that CSF-1R inhibition has the ability to inhibit MDSC polarization and activity, thereby sensitizing tumor cells to other forms of immunotherapy.

#### PI3Kγ

The phosphoinositide 3-kinase (PI3K) pathway is important in driving cellular proliferation and differentiation in both tumor and immune cells. PI3K can originally be activated in gliomas as a result of hypoxia, with downstream signaling resulting in recruitment of macrophages that can then be polarized to MDSCs (87). In the myeloid compartment, PI3K is expressed as PI3Kγ, which can be selectively targeted over PI3K expressed in other types of cells (88). Kaneda et al. demonstrated that PI3Kγ is crucial to immune suppression by activating nuclear factor kappa-light-chain-enhancer of activated B cells (NFκB) and inhibiting CCAAT-enhancer-binding proten beta (C/EBPβ) during macrophage polarization, resulting in an immunosuppressive phenotype (89). As a result, the group found that PI3Kγ knockout resulted in decreased tumor growth in several cancers. De Henau et al. found similar anti-tumor effects when 4T1 breast tumors and B16 melanomas bearing mice were treated with IPI-549, a myeloid-selective PI3Kγ inhibitor (90).

Within glioma cells, the PI3K pathway also has important effects that can potentially interact with the myeloid compartment. For example, PI3K has been implicated in radioresistance *in vitro* by Wang et al. (91), where PI3K inhibition resulted in increased radiation-induced apoptosis. In a later section, we see that the myeloid compartment is also involved in radioresistance, and thus non-selective PI3K targeting has the potential to improve outcomes of radiotherapy on multiple fronts. Quail et al. used non-selective PI3K blockade to overcome CSF-1R inhibition resistance, where the pathway has been implicated in a late resurgence of tumor growth following treatment with a CSF-1R inhibitor (86). In this case, the initial treatment effect was myeloid-based, but the resurgence was caused by signaling within the tumor, and addressing both sides resulted in improved survival. Considering these associations, PI3K inhibition has the potential to play a dual role by inhibiting myeloid immunosuppression and sensitizing the tumor to adjuvant therapies.

#### TYRO3/AXL/MERTK Receptor Tyrosine Kinases

TYRO3, AXL, and MERTK are a family of RTKs called TAM-RTKs and have been implicated in cell survival (92). Classically, their ligands protein S (PROS1) and growth arrest specific 6 (GAS6) are secreted by macrophages and cancer cells to activate PI3K, extracellular signal-regulated kinase (ERK), and NFκB pathways to promoted tumor proliferation and immune suppression (93). The role of TAM-RTKs in resistance to anticancer therapies have also been well-documented (94). Of note, shRNA knockdown of MERTK and AXL in G12 and A172 astrocytoma cell lines increased tumor apoptosis and autophagy pathways leading to increased chemo sensitivity to temozolomide and carboplatin (95). In mesenchymal GSCs, AXL was also found to be a key regulator of tumorgenicity and clonogenicity (96). Interestingly, AXL activation in head and neck squamous cell carcinoma cell lines resulted in increased expression of PD-L1 and radioresistance (97), providing rationale for potential synergy between AXL inhibition and anti-PD-1 therapy. In the context of the myeloid compartment, Ludwig et al. used a the novel AXL inhibitor BGB324 on a pancreatic cancer murine model and observed a prolonged median survival that was enhanced in combination with gemcitabine (98). The group also found that BGB324 treatment decreased MDSCs in the TME, suggesting that TAM-RTKs create an immunosuppressive environment by enriching the myeloid landscape with MDSCs.

#### COX-2

In various cancer types, COX-2 has been shown to push myeloid cells toward an immunosuppressive phenotype through prostaglandin E_2_ (PGE_2_) (99–101). Fujita et al. showed that COX-2 plays an important immunosuppressive role in gliomas as well (102). They found that COX-2 inhibition through acetylsalicylic acid (ASA) or celecoxib contributed to improved survival in C57BL/6J mice with implanted tumors derived from *SB de novo* gliomas. Total knockout of the COX-2 gene within the mice produced a similar result. ASA was further shown to decrease MDSC infiltration, lower the expression of CCL2; and increased influx of CTLs to the tumor site. Interestingly, COX-2 and CCL2 have been described as part of a positive feedback loop via prostaglandin E2 (PGE_2_), in which PGE_2_-mediated production of CCL2 induces COX-2 activity to produce more PGE_2_ (103). In this way, COX-2 promotes a pro-tumor response by immunosuppressive polarization as well as recruitment of MDSCs into the TME.

COX-2 inhibition has been combined with other myeloid targeted treatments to augment their anti-tumor response. For example, Kosaka, Ohkuri, and Okada found that combination treatment of GL261-bearing C57BL/6 mice with anti-CD40 agonist and celecoxib resulted in prolonged survival compared to monotherapies alone (21). Combination therapy resulted in decreased expression of *ARG1* in myeloid cells, a more robust CD4^+^ T cell activation, and a decrease in Tregs. In another potential combination, Kohanbash et al. showed that a subset of MDSCs in *SB*-induced gliomas express COX-2 (24). This expression was associated with the expression of other suppressive markers such as ARG1 and TGFβ. Expression of these markers could be induced by GM-CSF, which has been shown to have immunosuppressive properties in the setting of active tumor and chronic exposure but is also commonly used in immunotherapy for myeloid activation. Based on this result, targeting COX-2 could potentially enhance the efficacy of GM-CSF, as COX-2 inhibition can limit the immunosuppressive downstream effects of GM-CSF treatment, while preserving the myeloid-activating pathways. In fact, Eberstal et al. showed that in GL261 tumors, COX inhibitors improved survival in combination with GVAX, when compared to either treatment alone, as a result of greater T cell activation (63).

## Preclinical Studies of Combination Treatments Involving Myeloid-Targeting Therapy

Although the myeloid compartment is important in modulating the immune system, targeting myeloid cells alone is often not sufficient to elicit an effective immune response. As a result, combination therapies are often necessary to achieve desired treatment outcomes. Additionally, many existing treatment modalities affect and are affected by the myeloid compartment, therefore, emphasizing the need for combination with myeloid targeting to prevent myeloid-mediated therapy resistance. A summary of the following preclinical studies is available in Table 1.

**Table 1:** Preclinical studies of combination treatments targeting myeloid cells.

**Myeloid treatment**	**Treatment effect**	**Combination therapy**	**Cancer type and reference**	**Synergistic effects**
Alisertib	MDSC depletion	Oncolytic virus	Malignant peripheral nerve sheath tumor (104)	Oncolytic virus causes tumor lysis and myeloid recruitment, alisertib causes MDSC depletion
All-trans retinoic acid	Decrease level of MDSCs	CAR-T cells	Sarcoma (105)	Decrease in MDSCs lowers inhibition of CAR-T cells
Axitinib	VEGF inhibition, increase myeloid infiltration, reduce suppressive capacity	anti-CTLA-4	MO4 tumors (106, 107)	Axitinib allowed for more stimulatory TME, which allowed ICI to be more effective
CCL2 blockade	Prevention of MDSC polarization	TMZ	Glioma (30)	Both result in decrease in MDSCs
CD40	Activation of myeloid dendritic cells	ICI + GVAX	PDAC (59)	See above for ICI + GVAX effect. CD40 improves efficacy of antigen presentation
		Sunitinib	Melanoma and fibrosarcoma (108)	Sunitinib causes MDSC depletion, while CD40 activates antigen presentation in myeloid DCs
COX-2 inhibition	Prevention of MDSC polarization	Sunitinib	Renal cancer (109)	Sunitinib causes MDSC depletion, while COX inhibition prevents further MDSC polarization
CSF-1R inhibition	Prevention of MDSC polarization	ICI + GVAX	PDAC (110)	See below for ICI + GVAX effect. CSF-1R decreases myeloid suppression
		ICI + DC vaccination	Glioma (111)	CSF-1R decreases myeloid suppression while DC vaccination and ICIs promote targeted T cell response
		Topotecan	Neuroblastoma (112)	Decrease in MDSCs enhances efficacy of chemotherapy
Gemcitabine	MDSC depletion	Oncolytic virus	ID8 (113), colorectal cancer (114)	Oncolytic virus causes tumor lysis and myeloid recruitment, gemcitabine causes MDSC depletion
GM-CSF	Antigen uptake and presentation by myeloid cells	Oncolytic virus	Glioma and melanoma (115), colorectal cancer (116), breast cancer (117)	Oncolytic virus causes tumor lysis and myeloid recruitment, GM-CSF promotes DC differentiation
GVAX/GM-CSF vaccination	Antigen uptake and presentation by myeloid cells	ICI	Glioma (57), bladder cancer (58), PDAC (59)	GVAX allows for a more targeted T cell response while ICI blocks immune checkpoint
		OX40 activation	Glioma (118)	GVAX allows for a more targeted T cell response while OX40 increases T cell activation
		Radiation + Agonist anti-CD137	Glioma (62)	Increased myeloid activation allows for better response to radiation-weakened tumor. CD137 agonist enhances T cell response
IL-12	Myeloid (and T cell) activation	ICI	Glioma (78, 79, 119)	IL-12 inhibits myeloid suppression (and promotes T cell activation) while ICI blocks immune checkpoint
		Oncolytic virus	Sarcoma (120), glioma (79, 119)	Oncolytic virus causes tumor lysis and myeloid recruitment, IL-12 promotes myeloid activation
Macrophage phagocytosis agonist	Activation of macrophages	TMZ	Glioma (121)	Undetermined
Magnetic nanoparticles	Prevention of MDSC polarization	Radiation	Glioma (122)	Decrease in MDSCs results in decreased radioresistance
PGE2 targeting	Prevention of MDSC polarization	Oncolytic virus	Variety of tumors (123)	Oncolytic virus causes tumor lysis and myeloid recruitment, PGE2 targeting prevents MDSC polarization
PI3K inhibition	Prevention of MDSC polarization	ICI	Breast cancer and melanoma (90)	PI3K inhibition decreases myeloid suppression while ICI blocks immune checkpoint
siGal-1	Decrease level of MDSCs	ICI	Glioma (124)	Decrease in MDSCs allows for more effective checkpoint blockade
		DC vaccination	Glioma (48)	Decrease in MDSCs allows for more effective DC vaccination promotes targeted T cell response from DC vaccination
		TMZ	Glioma (124)	Both result in decrease in MDSCs
STING agonist	Increase in myeloid (and other) DCs	ICI + OX40 activation	Breast cancer (70)	STING agonist allows for stronger T cell response resulting from other therapies
STING agonist + CCR2 blockade	Decrease in MDSCs and increase in myeloid DCs	Radiation	Colon cancer (71)	Myeloid therapy decreases myeloid suppression, thereby decreasing radioresistance
TAM-RTK blockade	Prevention of MDSC polarization	ICI	Glioma (125)	TAM-RTK inhibition decreases myeloid suppression while ICI blocks immune checkpoint
Toca 511/Toca FC	Depletion of MDSCs	Toca 511/Toca FC	EL4 (126), glioma (127, 128), colorectal cancer (129)	Toca causes depletion of MDSCs and both cause tumor cell death
		Chemotherapy (TMZ or lomustine)	Glioma: TMZ (130), lomustine (131)	Toca causes depletion of MDSCs and tumor cell death
		Radiation	Glioma (132)	Toca causes decrease in radioresistance
Tumor peptide vaccination	Antigen uptake and presentation by myeloid cells	Sunitinib	Melanoma (133), TC1 (134)	Depletion of MDSCs allows for more effective vaccination

### T Cell Therapies

Immune checkpoints under normal conditions provide co-stimulatory and co-inhibitory signals to modulate T cell immune responses. In the setting of cancer, tumors can manipulate ICs by expressing inhibitory receptors or upregulating T cell expression of inhibitory ligands to blunt the body's normal anti-tumor immune response. Inhibitory antibodies to PD-1, cytotoxic T-lymphocyte-associated protein 4 (CTLA-4), and lymphocyte-activation gene 3 (LAG3) have emerged as strategies to facilitate enhanced anti-tumor response and have shown efficacy in systemic cancers such as melanoma and non-small cell lung cancer. Their effects have been less dramatic in cold tumors such as GBM. This is likely due to the presence of alternative tumor-induced inhibitory pathways. Previous work has shown that MDSCs in the TME express immune checkpoint molecules and contribute to the inhibition of CTL function and maintenance of Tregs, which ultimately exert a suppressive effect on TILs (135, 136). The presence of MDSCs therefore acts as an escape mechanism by which the tumor can overcome immune checkpoint blockade. Impeding the recruitment of MDSCs or re-educating the myeloid compartment to a more immunostimulatory phenotype as supplemental therapy to checkpoint blockade has been shown in preclinical models to enhance anti-tumor effects.

The combination of GVAX to prime the myeloid compartment and anti-CTLA-4 checkpoint blockade to disinhibit TILs has been well-characterized in murine melanoma models and patients with metastatic melanoma (60). Agarwalla et al. have conducted the first reported study combining GVAX and anti-CTLA-4 therapy in an intracranial GL261 glioma mouse model and concluded that sequential injection of irradiated GL261 cells expressing GM-CSF followed by anti-CTLA-4 therapy significantly prolonged survival compared to individual monotherapies (57). In a similar fashion, Zhang et al. recently reported therapeutic success in combining anti-PD-1 checkpoint blockade with an anchored GM-CSF vaccination in MB49 bladder cancer (58). The synergistic effect of combining myeloid based therapy and immune checkpoint blockade is likely resulting from activation of TILs with GM-CSF followed by anti-PD-1 treatment negating functional immunosuppression from tumor PD-L1 expression. Ma et al. has recapitulated these findings in murine models of pancreatic ductal adenocarcinoma (PDAC) and breast cancer, two solid-tumor cancers that are known to have limited immunogenicity (59). Triple therapy with agonist anti-CD40, a 3T3 fibroblast analog of GVAX (3T3neuGM), and anti-PD-1 significantly shifted the myeloid compartment from MDSCs to activated DCs and led to increase TIL infiltration. In gliomas, Jahan et al. (118) found that agonist anti-OX40 immunotherapy enhances activity of activated lymphocytes and works synergistically with GVAX against an intracranial glioma model in C57BL/6 mice. Combination therapy resulted in improved survival as well as improved T cell infiltration and anti-tumor function. The same group added to this by using a triple therapy regimen of GVAX, anti-PD-1, and agonist anti-OX40 in GL261 glioma-bearing mice, demonstrating 100% long-term survivorship (137). Dual therapy with GVAX and anti-PD-1 also significantly prolonged median survival, with an observed 50% long-term survival rate. Here again we see that GVAX likely stimulated the myeloid compartment toward an activated, antigen presenting phenotype, while the OX40 agonist promoted activation of T cells (138).

The concept of using colony stimulating factors to stimulate or target the myeloid compartment is multi-faceted. While GM-CSF signaling through GM-CSF receptor 2 (CSF-2R) contributes to macrophage polarization into DCs, M-CSF signaling through CSF-1R shapes the myeloid landscape into an immunosuppressive phenotype (139). Since Antonios et al. found that DC vaccination increases the amount of PD-L1-expressing MDSCs in the TME, the group hypothesized that CSF-1R blockade with anti-PD-1 therapy would enhance anti-tumor effects (111). In a murine GL261 glioma model, triple therapy with CSF-1R blockade, anti-PD-1 therapy, and DC vaccine conferred a 50% long-term survival rate and prolonged median survival compared to double therapies. Their proposed model for triple therapy argues that DC vaccination ultimately stimulates TIL infiltration but does not address TIL inactivation via PD-1/PD-L1 signaling. To this end, CSF-1R inhibition in combination with anti-PD-1 therapy further endorses an immunostimulatory environment by converting MDSCs to pro-inflammatory myeloid cells and by inhibiting PD-1-mediated T cell inhibition. Saung et al. also found that administration of GVAX and anti-PD-1 therapy in a PDAC mouse model upregulated CSF-1 expression in the TME and resulted in increase of MDSCs (110). Of note, treatment with anti-CSF-1R both prior to and after GVAX and anti-PD-1 was necessary for synergistic anti-tumor effects. Using anti-CSF-1R exclusively before or after the other treatments resulted in fewer T cell infiltration. This indicates that persistent inhibition of the CSF-1 axis is needed for myeloid cells to remain in an immunostimulatory state.

The PI3Kγ axis has been recognized as a critical component of tumor recurrence and of polarization of macrophages to the immunosuppressive phenotype. In fact, other myeloid targets, such as COX-2 (140) and CSF-1R (141) exert their pro-tumoral effects through upregulation of expression of PI3Kγ in macrophage and microglia. De Henau et al. used a novel macrophage-targeting PI3Kγ inhibitor IPI-549 in combination with anti-PD-1, and anti-CTLA-4, which resulted in prolonged median survival and long-term survival in breast cancer and melanoma murine models (90). The group also demonstrated that IPI-549 polarized myeloid cells to an anti-tumor phenotype, subsequently increasing the CTL/Treg ratio *in vivo*.

vom Berg et al. utilized a different approach in mice implanted with GL261 glioma cells by inhibiting the suppressive activity of MDSCs through IL-12 (78). Interestingly, IL-12 therapy was found to prolong survival in an IFNγ-independent fashion and resulted in upregulation of CTLA-4 in TILs. Although local delivery of IL-12 via osmotic minipump markedly prolonged median survival, combination with systemic CTLA-4 blockade resulted in full remission in 80% of treated mice. As a follow-up to this study, and to assess the efficacy of IL-12 on GSCs, which are involved in polarization of myeloid cells toward immunosuppressive phenotypes, Saha et al. engineered a G47Δ strain of oncolytic herpes virus (oHSV) to express mouse IL-12 (oHSV G47Δ-mIL-12) (119). Combination of oHSV G47Δ-mIL-12 with either anti-PD-1 or anti-CTLA-4 therapy corroborated with vom Berg et al's findings and conferred a moderate increase in median overall survival. More significantly, triple combination with oHSV G47Δ-mIL-12, anti-PD-1, and anti-CTLA-4 therapy virtually eliminated the GSC tumors in mice and conferred universal long-term survival upon reinoculation with GSCs.

Since TAM-RTKs have been characterized to block IL-12 production (142) and facilitate immunosuppressive polarization (94), Sadahiro et al. have targeted the AXL arm of TAM-RTKs as a potential therapy in mice implanted with mesenchymal GSC-derived tumors (125). The authors used the small molecule AXL inhibitor BGB324 in combination with anti-PD-1 checkpoint blockade and found a moderate increase in median survival compared to the control group with long term survival in about 10% of treated mice. They also found that AXL activation in the TME is mediated through increased expression of PROS1, a known TAM ligand that was originally thought to only bind to TYRO3 and MERTK. Furthermore, anti-PD-1 therapy was observed to increase AXL expression and levels of CD11b^+^ myeloid cells in the TME, confirming the rationale of using an AXL inhibitor in combination with checkpoint blockade.

To address Gal-1-mediated regulation of MDSCs and subsequent therapy resistance in glioma, Van Woensel et al. treated mice implanted with GL261 glioma cells with anti-Gal-1 siRNA (siGal-1) and anti-PD-1 therapy (124). siGal-1 treatment was shown to decrease the pool of M-MDSCs and Tregs in the TME. Co-staining also revealed an increase in infiltrating T cells in the TME. The group found that siGal-1 works synergistically with anti-PD-1 treatment, almost doubling median survival (30 days in anti-PD-1 arm vs. 51.5 days in the combination arm) with a 20% long-term survival rate. Verschuere et al. (48) showed that Gal-1 knockdown also worked synergistically with DC vaccination to result in long-term survival in 34% of inoculated mice. The immunopermissive niche allowed by the Gal-1 knockout likely prevented suppression of the T cells activated by DC vaccination, enabling them to properly mount an anti-tumor response. Gopinath et al. targeted downstream effectors of Gal-1 by disrupting the CCL2-CCR2 axis in gliomas to target MDSC trafficking to the TME (143). Using the CCR2 antagonist CCX598, the group showed that while monotherapy did not prolong survival in GL261 tumor-bearing mice, the addition of anti-PD-1 or anti-PD-L1 treatment slowed glioma progression. In a similar fashion, Highfill et al. demonstrated that rhabdomyosarcoma (RMS) utilizes the IL-8/CXCR1/2 axis to recruit MDSCs (144). The group showed that, while CXCR2-knockout mice did not consistently exhibit increased survival compared to wild-type mice when implanted with RMS cell lines, combination therapy with anti-PD-1 checkpoint blockade resulted in markedly increased median survival in all cell lines, as well as long-term survivorship in one cell line.

Combination of multiple myeloid targeted therapies in addition to lymphocyte targeted therapies has also been shown to have synergistic effects on the anti-tumor response. In FVB mice with breast tumors expressing human epidermal growth factor receptor 2 (HER-2), Foote et al. used a STING agonist to improve the efficacy of combination treatment with anti-PD-L1 and OX40 agonist by increasing activation of myeloid DCs, priming tumor-specific CTLs, and abrogating immunosuppressive signals (70). In this treatment, the STING agonist allowed for a more immunopermissive TME with enhanced antigen presentation, while OX40 increased T cell activation, rendering anti-PD-L1 therapy much more effective.

In addition to immune checkpoint blockade, targeting the myeloid compartment has also been shown to boost the anti-tumor effect of CAR T cell therapies. Long et al. showed that treatment of NOD scid gamma (NSG) mice in a sarcoma model with all-trans retinoic acid, which is known to promote differentiation of immature myeloid cells into immunostimulatory phenotypes, resulted in a loss of monocytic MDSCs and loss of suppressive function in granulocytic MDSCs (105). Combination treatment of all-trans retinoic acid with CAR-T cells improved survival. Interestingly, the CAR-T cells expressing OX40 and CD28 receptors, both of which are involved in T cell activation, were more effective than CAR-T cells that did not express both receptors, highlighting the importance of multiple modes of activation.

### Oncolytic Viral Therapy

Oncolytic viruses are viruses that preferentially kill tumor cells and also enhance the induction of anti-tumor immunity that accompanies the oncolytic activity. Therefore, a combination of oncolytic viral therapy with other immune based therapies targeting the myeloid compartment can enhance the efficacy of oncolytic viral therapies. Several different types of virus have been used for their specificity in targeting cancer cells. For example, Liu et al. have created an oHSV strain for intratumoral injection, in which a neurovirulence factor has been inactivated, resulting in tumor-specific infection in murine gliomas and melanomas (115). Upon the insertion of the GM-CSF gene into the virus, the group showed an increase in activation of splenocytes *in vitro*, correlated with greater tumor shrinkage *in vivo*. In a clinical trial involving patients with metastatic melanoma treated with GM-CSF-transfected oHSV, Kaufman et al. also showed a decrease in MDSCs in vaccinated lesions (145). Yin et al. demonstrated a similar enhanced effect in colorectal cancer with a different type of GM-CSF-expressing oHSV, which resulted in decreased MDSC infiltration and an increase in local mature DCs (116). oHSV has also been engineered to express IL-12, which was demonstrated by Ring et al. to decrease infiltrating MDSC levels in sarcomas more so than oHSV alone (120). While oncolytic viruses can elicit anti-tumor immune response through antigen release after tumor cell death, they are also subject to targeting by circulating antibodies and host immune response, therefore leading to recruitment of immune cells to the injected tissue. Currier et al. demonstrated that oHSV can induce accumulation of MDSCs in the TME (104). This phenomenon was reversed by alisertib, a serine/threonine-protein kinase 6 inhibitor, in malignant peripheral nerve sheath tumors. Combination of oHSV and alisertib lead to a synergistic decrease in tumor growth. In support of these results, Esaki et al. also showed enhanced anti-tumor response by enhancing oHSV therapy with myeloid depletion therapy gemcitabine in colorectal cancer models (114). These studies highlight the synergistic anti-tumor response of combining oncolytic viral therapy with myeloid targeted therapies.

Vaccinia virus has also been used for its oncolytic properties. Similar to oHSV, vaccinia expressing GM-CSF has been used by de Vries et al. to enhance anti-tumor immunity in breast cancer (117). Vaccinia has also been engineered by Hou et al. to target PGE_2_, which, as described previously, is involved in MDSC polarization (123). This resulted in improved survival over treatment with viruses without PGE_2_ targeting capability. It has been shown that vaccinia virus can attract both immunostimulatory myeloid cells and MDSCs to the site of injection. Tan et al. showed that treatment with vaccinia viral therapy alone led to infiltration with MDSCs that suppressed DC function (146). On the other hand, Kilinc et al. (147) found an increase in activated myeloid cells upon treatment with vaccinia. Thus, it may be advantageous to deplete the suppressive MDSCs that are induced by vaccinia, while preserving stimulatory myeloid cells, highlighting the need for combination therapies. In fact, Liu et al. circumvented this issue after finding a large amount of PD-L1 expressing MDSCs in the tumor after vaccinia treatment by treating with anti-PD-L1, improving survival and decreasing tumor burden (148).

Reovirus is another commonly used oncolytic virus that has been shown to impact the myeloid population in the TME. Clements et al. demonstrated that in ovarian cancer, reovirus increases intratumoral MDSCs and expression of immunosuppressive genes (149). Katayama et al. found that reovirus inhibited the T cell suppressive function of MDSCs through toll-like receptor 3 (TLR3), which was abrogated in a TLR3 knockout model (150). Furthermore, Gujar et al. found that the anti-tumor effect of reovirus therapy was enhanced by MDSC depletion with gemcitabine (113).

Other viruses have also been used for their oncolytic properties. In colon carcinoma, Scherwitzl et al. showed that treatment with Sindbis virus expressing tumor-specific antigen in colon carcinoma lead to anti-tumor immune responses (151). Combination therapy of Sindbis virus with PD-1 blockade resulted in significantly improved overall survival and reduced MDSC infiltration in the TME. Another oncolytic strain is Newcastle disease virus, which Koks et al. found that treatment with another oncolytic virus, Newcastle disease virus, resulted in increased T cell activation, MDSC depletion, and improved survival in gliomas (152). Finally, in a creative treatment, Eisenstein et al. (153) found that MDSCs can be used as a vehicle for oncolytic rhabdovirus. MDSCs infected with the virus and transferred to the host not only lead to a localized infection at the tumor, but also assumed a more immunostimulatory phenotype.

In summary, oncolytic viruses appear to recruit many types of myeloid cells into the tumor, both immunosuppressive and immunostimulatory. Combination treatments are most effective upon inhibition of the suppressive myeloid cells while preserving those that enhance anti-tumoral immune responses.

### Combination Myeloid-Targeting Therapies

In addition to the success of combining myeloid targeted therapy with immunotherapy aimed at boosting other aspects of the immune system, combination therapies targeting multiple immunosuppressive myeloid pathways have also shown some promise. However, it is important to consider that altering only the myeloid compartment, even through multiple pathways, may not suffice for a full anti-tumor response. It has been shown previously that while GM-CSF can activate the immune system, it can also upregulate suppressive factors in myeloid cells such as COX-2 (24). As previously mentioned, Eberstal et al. combined GVAX with COX-2 inhibition via systemic administration of parecoxib and intratumoral administration of valdecoxib, demonstrating that inhibition of COX-2 enhances the efficacy of GVAX and improves survival (63). Kosaka et al. used the COX-2 inhibitor celecoxib in concert with CD40 to inhibit MDSC polarization, promote DC differentiation, and increase T cell activation (21). Finally, Chen et al. used triple therapy with intratumoral delivery of GM-CSF, IL-12, and irradiated tumor vaccine in the treatment of gliomas (154). These therapies acted on different components of the myeloid cells, with irradiated tumor vaccines promoting antigen presentation, IL-12 inhibiting MDSC polarization and promoting DC maturation, and GM-CSF promoting growth of the antigen-presenting myeloid cells. The authors found that each dual therapy combination enhanced survival in gliomas with triple therapy leading to the longest survival benefit. However, it should be noted that IL-12 also has significant effects on the CTL populations, which would be necessary for an effective immune response.

### Molecular Therapies

Molecular targeted therapies have also been used in combination with myeloid targeted therapies to modulate the myeloid compartment in anti-cancer treatments. RTK inhibitors, for example, can inhibit VEGF, which can lead to MDSC differentiation and recruitment to the TME. Sunitinib, a RTK inhibitor, has been shown to reduce MDSC levels in human renal cell carcinoma (155). Several groups have taken advantage of this, thereby combining sunitinib treatment with myeloid targeting therapies. van Hooren et al. demonstrated that sunitinib synergistically enhanced treatment of B16 melanomas and T24 fibrosarcomas with anti-CD40 agonist antibodies (108). Sunitinib resulted in a decrease in MDSCs while anti-CD40 increased DC activation. Zhao et al. (109) used sunitinib in conjunction with celecoxib to drastically inhibit tumor growth. In this case, both modalities worked to decrease the level of MDSCs. Bose et al. was able to enhance the efficacy of tumor-specific peptide-pulsed DC vaccine with sunitinib, showing that combination treatment had a lower level of MDSCs (133). This effect was mirrored when Draghiciu et al. showed lower levels of MDSCs and increase in CTLs when combining sunitinib with a viral vaccine for HPV-induced oncoproteins (134).

Another RTK inhibitor, sorafenib, has shown promise as well in promoting anti-tumor immune responses. Heine et al. has shown that sorafenib leads to decreased MDSC immunosuppressive capacity *in vitro*. However, the effect of sorafenib appears to be dependent on MDSC levels (156). Chang et al. showed that the treatment efficacy of sorafenib was decreased in tumors with high levels of MDSCs, but that upon antibody-mediated depletion of MDSCs, the efficacy of sorafenib was restored (157). This suggests that combining sorafenib with an MDSC-depleting therapy could result in a synergistic effect. Axitinib, a VEFGR inhibitor, has also been shown to increase myeloid infiltration while simultaneously reducing the suppressive capacity of MDSCs (106). This could prime the TME into an immunostimulatory state, allowing for improved efficacy of additional immune based therapies. In fact, Du Four et al. combined axitinib with CTLA-4 blockade and found that combination treatment synergistically reduced tumor growth and improved survival (107).

Cetuximab is an antibody against EGFR that has been shown to impact myeloid function and phenotypes. EGFR is a commonly mutated gene in multiple cancers has served as a common molecular target. Li et al. hypothesized that the Fc portions of cetuximab may interact with the Fcγ receptor on myeloid cells to alter their phenotype (158). This hypothesis was further supported by a recent clinical trial (NCT01218048) that analyzed blood from head and neck squamous cell carcinoma patients treated with cetuximab and showed polarization of myeloid cells toward an immunostimulatory phenotype. Jia et al. then showed in mice that EGFR molecular inhibitors also cause an increase in immunostimulatory myeloid cells (159). However, this effect was transient, lasting only the length of treatment, and was counteracted by alterative immunosuppressive pathways involving CCL2, ultimately leading to a persistent increase in MDSCs. The authors suggested that a combination of CCL2 and EGFR inhibition could increase anti-tumor effects compared to EGFR inhibitors alone.

### Radiation

While immunotherapy has been integrated into the treatment regime of several systemic cancers, radiation, and chemotherapy remains the main stay of treatment for various tumors, especially gliomas. Therefore, it is crucial to understand the effects of standard adjuvant therapies on the anti-tumor immune response and vice-versa to optimally integrate these novel therapies into current standard of care. The effect of radiation on the myeloid population is 2-fold. It has been shown to increase tumor infiltration of both immunosuppressive and immunostimulatory populations of myeloid cells (160). Furthermore, radiation-induced necrosis and apoptosis of tumor cells also lead to release of tumor antigens and antigenic spread leading to enhanced tumor-specific immune responses (161–163). In an MC38 colon cancer model, Liang et al. showed that radiation treatment resulted in increased levels of MDSCs, which subsequently contributed to radiation resistance developed by the tumors (71). Interestingly, this radioresistance was abrogated by CCR2 blockade. The group further showed that combination therapy with radiation and CCR2 blockade resulted in improved treatment outcomes when compared to radiation alone. Similarly, combination therapy with a STING agonist, cGAMP, CCR2 blockade, and radiotherapy resulted in lower MDSC infiltration and decreased tumor volume.

In the setting of gliomas, Newcomb et al. showed in a GL261 glioma models that treatment with radiotherapy in combination with GVAX resulted in improved survival over either treatment alone (62). The group theorized that GVAX was able to prime the myeloid population toward a more immunostimulatory state, rendering the tumors more sensitive to radiotherapy. The same group also showed that radiation enhances the antitumor effect of agonist anti-CD137 antibody therapy. Combination-treated mice observed increased TILs in the TME, as well as prolonged survival compared to control and monotherapy groups. CD137 (4-1BB) has been implicated in differentiation of monocytes into DCs (164) and activation of CD4^+^ and CD8^+^ T cells leading to increased anti-tumor responses (165).

Nanoparticles that target MDSCs to promote polarization of myeloid cells to an anti-tumor phenotype have also been used in combination with radiotherapy with promising effects. In CT2A and U87 gliomas, Wu et al. treated mice with magnetic nanoparticles, aimed at targeting both MDSCs and tumor cells directly, along with radiation therapy (2 Gy/day for 4 days) 7 days post-tumor implantation (122). They showed increased median survival with the combination therapy when compared to radiation alone. The combination therapy pushed the myeloid compartment into an anti-tumor phenotype and increased the expression of immunostimulatory genes such as tumor necrosis factor alpha (TNFα) and inducible nitric oxide synthase (iNOS). In addition to radiosensitizing tumor cells and modulating the TME, nanoparticles can also be readily uptaken by myeloid cells such as macrophages. Myeloid cells can then traffic the phagocytosed nanoparticles to the TME where they can lead to anti-tumor response and MDSC repolarization (166). Better understanding of the interplay between nanoparticles, MDSCs, and radiotherapy will be necessary to optimally combine these therapies to boost anti-tumor response. With continued evolution of radiotherapy aimed at increasing targeted radiation dose to the tumor while minimizing collateral damage to surrounding normal tissue, other types of radiation such as carbon irradiation (CIR) and proton irradiation (PIR) have gained popularity over traditional photon irradiation. It has been shown that different types of radiation can affect the myeloid compartment differently. Chiblak et al. showed CIR to be more beneficial than standard PIR in immunotherapy in several key areas (167). They showed that treatment with CIR led to decreased MDSC infiltration and an increase in pro-inflammatory myeloid cells compared to PIR. *In vitro*, microglial migration was reduced with CIR and increased with PIR. Monzen et al. showed that CIR can inhibit the growth of MDSCs and their progenitors over PIR, which could partially explain the treatment advantage of CIR over PIR in the context of immunotherapy (168).

### Chemotherapy

Currently, the most commonly used chemotherapeutic in GBM is temozolomide (TMZ), an alkylating antineoplastic drug that causes cytotoxicity through guanine and adenine methylation (169). Mathios et al. demonstrated that synergistic effects can exist between locally-delivered TMZ and immunotherapy (170). Unfortunately, systemic TMZ, which is the current standard of care, results in immunodepletion and thus can limit the efficacy of immune-based therapies. In the case of MDSCs, immunodepletion could be advantageous, as several of the following groups have shown.

In GL261 glioma and human U87 glioma xenograft mouse models, Zhu et al. showed that CCL2 antibody blockade in combination with TMZ resulted in improved survival over either monotherapy alone (30). As discussed previously, CCL2 binds to CCR2 and results in the recruitment of TAMs and polarization to MDSCs (171).

Van Woensel et al. demonstrated that treatment with siGal-1 can lower the presence of MDSCs and Tregs in GL261 tumors (124). Knockdown of Gal-1 also resulted in more normalized vasculature that allowed for greater penetration by TMZ leading to more effective tumor killing. In BALB/c nude mice and C57BL/6 mice subcutaneously injected with U87 and GL261 cells, respectively, Zhang et al. (172) showed that combination treatment with an agonist for macrophage-mediated phagocytosis (SIRPα-Fc), an autophagy inhibitor (chloroquine), and TMZ resulted in significantly prolonged survival compared to control, monotherapy, and double therapy groups.

In another study, Webb et al. treated a patient-derived neuroblastoma xenograft in T cell deficient mice with a combination of the CSF-1R inhibitor BLZ945 and the chemotherapeutic topotecan (112). They showed a decrease in myeloid cells with BLZ945 alone, but no effect on survival. However, upon addition of chemotherapy, there was an increase in survival with combination therapy over chemotherapy alone. It is clear from these results that myeloid cells can have an inhibitory effect on chemotherapy independent of T cell function and inhibition of myeloid immunosuppression can improve chemotherapy outcomes.

Another creative chemotherapy with effects on the myeloid compartment utilizes the retroviral vector Toca 511. With this technique, Toca 511 selectively delivers a cytosine deaminase gene to cancer cells. Cytosine deaminase is then expressed by the infected cells, causing them to convert the pro-drug 5-fluorocytosine (5-FC), commonly delivered in the oral extended-release form of Toca FC, into the potent chemotherapeutic 5-fluorouracil (5-FU), which causes death of the tumor cells (173). Besides tumor cell death, 5-FU has also been shown by Vincent et al. to selectively kill MDSCs in tumor cells while preserving other immune populations and resulting in greater T cell IFNγ production (126). Mitchell et al. then confirmed this effect in the context of Toca by pretreating tumor cells before flank implantation with Toca 511, followed by treatment with Toca FC (127). Intratumoral injection of Toca 511 in gliomas by Hiraoka et al. (128) and colorectal cancer by Yagiz et al. (129) also resulted in immunological benefit. The immunological effects were correlated with survival and long-term resistance to tumor rechallenge. Survival effects were preserved on combination therapy with both TMZ (130) and lomustine, another chemotherapeutic (131). Additionally, Takahashi et al. demonstrated a decrease in radioresistance caused by treatment of gliomas, although this was done in athymic mice, and thus the possible immunological contribution is unclear (132). The success of Toca supports the findings of Mathios et al. where localized chemotherapy was beneficial for cultivating an immune response (170). The exact mechanism of the synergistic anti-tumor effects of combining myeloid targeted therapies with chemotherapy is unclear. It has been postulated that chemotherapy, similar to radiation therapy, generates new antigenic targets and boosts antigenic uptake and presentation thereby priming the TME for adaptive anti-tumor immune responses. Combination with myeloid targeted therapies can further abrogate alternative immunosuppressive pathways to enhance anti-tumor effects of chemotherapy.

### Corticosteroids

An adjunct treatment that is unique to the treatment of gliomas and other brain tumors is corticosteroids. Steroids such as dexamethasone are used to decrease cerebral edema caused by the tumor and has been shown to lead to significant alterations to the immune compartments. Maxwell et al. previously showed that administration of steroids led to decreased peripheral CD4^+^ and CD8^+^ T cells and led to decreased efficacy of anti-PD-1 treatment for peripheral flank tumors compared to intracranial tumors (174). While historically, steroids were thought to affect the lymphocyte population, studies now have shown similar effects of steroids on the myeloid compartment as well. Moyes et al. has demonstrated that treatment with dexamethasone resulted in an increase in peripheral circulator myeloid populations (175). As we continue to elucidate the mechanism of immunosuppression caused by steroids in the context of cancer immunotherapy, it is important to account for their potential effect on the myeloid compartment in addition to lymphocyte populations.

## MDSCs as Predictive Biomarkers

In addition to identifying the therapeutic benefits of myeloid-based therapies, characterizing the myeloid compartment offers the potential for stratifying patient prognosis and response to immunotherapy by measuring myeloid-specific biomarkers. Other biomarkers such as tumor mutational burden, checkpoint expression, and T cell receptor diversity have been used to predict response to immunotherapies, where increases in each are correlated with improved patient outcomes (176). In gastric cancer, higher levels of TAMs have been associated with increased tumor progression (177), which could be inferred from the immunosuppressive functions of tumoral myeloid cells. Circulating MDSC levels in patients have also been used as a biomarker for response to immunotherapies in patients with melanoma, colorectal, kidney, prostate, and breast cancer, with increases in blood MDSCs correlating with worse patient outcomes (178–180). Alban et al. found a similar correlation in GBM, with higher circulating MDSCs associated with worse prognosis and survival (181). Additionally, in melanoma patients, Huber et al. found that microRNAs (miRNAs) through which tumors induce MDSC formation can be used as a predictive biomarker of response to immunotherapy (182). The authors found that clustering patients based on the quantity of intratumoral MDSC miRNA stratified patients' response to ICI therapy. These studies indicate that myeloid-based characterizations have the potential to serve as biomarkers of outcome and treatment response to identify patients who are most likely to respond to a particular therapy.

## Clinical Trials

Although the majority of preclinical evidence for combination treatments involving various immune based and cytotoxic cancer therapies along with myeloid targeted therapies has shown promise, targeting myeloid cells for immunotherapy is a fairly recent endeavor. As a result, many clinical trials using combination treatment with myeloid therapies are still ongoing, with results yet to be reported. Some encouraging results have emerged from a few published trials. In a Phase I trial (NCT02526017) of combination therapy of cabiralizumab, a CSF-1R blocking antibody, with nivolumab (anti-PD-1) in a variety of solid tumors showed significant depletion of TAMs. They further demonstrated a tolerable safety profile and durable clinical benefit, with response in 5 of 31 advanced pancreatic cancer patients (183). Based on this trial, a Phase II trial in of cabiralizumab and nivolumab in combination with chemotherapy is underway for advanced pancreatic cancer (NCT03336216).

GM-CSF has also been investigated in the clinical setting as a combination therapy. A Phase II trial in GBM (NCT01498328) utilizing GM-CSF with an EGFRvIII peptide vaccine and bevacizumab has recently completed. The combination treatment showed efficacy over bevacizumab alone, as measured by overall response rate (ORR) and progression free survival (PFS), confirming its efficacy (184). Similar preliminary results have been reported in an ongoing GBM Phase II trial of ERC1671, or Gliovac, which consists of inactivated tumor cells and tumor lysate, in combination with GM-CSF, cyclophosphamide, and bevacizumab, with vaccinated patients surviving longer than non-vaccinated, bevacizumab treated counterparts (185). GVAX has also been evaluated in combination with pembrolizumab in a Phase II trial in metastatic colorectal cancer. While the treatment was well-tolerated, treatment outcomes were comparable to historical control (186).

PI3Kγ is also being targeted via the aforementioned selective inhibitor, IPI-549. In a Phase I trial in several tumor types (NCT02637531), treatment with IPI-549 in combination with nivolumab has so far been shown to be tolerable. Preliminary results have shown some immunological effects, including a decrease in immunosuppression and increased T cell proliferation (187).

Acknowledging the role of IL-8 in MDSC recruitment, Collins et al. recently conducted a Phase I clinical trial with HuMax-IL8, an anti-IL-8 monoclonal antibody, to assess the safety profile and efficacy of this therapy in reducing serum IL-8 levels in patients with solid tumors (NCT02536469). Their trial concluded that IL-8 blockade was well-tolerated and successfully decreased serum IL-8 levels in subjects across all doses tested (188). Given the results of this clinical trial, combination with ICI may be underway.

STING agonist MK-1454 is being used intratumorally in combination with systemic pembrolizumab in treatment of solid, glioma tumors and lymphomas in an ongoing Phase I trial (NCT03010176) with reports of partial response in a number of patients (189). In two other Phase I trials with similar tumors, the STING agonist ADU-S100 is being tested in combination with ipilimumab (anti-CTLA-4, NCT02675439) and PDR-001 (anti-PD-1, NCT03172936). A majority of the patients have dropped out of the STING monotherapy arm due to disease progression. However, lesion biopsies have shown an increase in tumor-infiltrating CD8^+^ T cells, indicating some immunological effect with combination therapy (190).

T-VEC, an oncolytic herpes simplex virus that expresses GM-CSF, has been FDA approved for treatment of melanoma. Since approval, a number of trials have attempted to use this therapy in combination with other immune-based therapies. Notably, a Phase Ib/II trial of T-VEC combined with ipilimumab (NCT01740297) has shown promising results, with a doubling of ORR when compared to ipilimumab alone (191). Pexa-vec, a vaccinia virus also engineered to express GM-CSF, is also the subject of a number of clinical trials of combination treatments.

Finally, the results from a completed Phase I trial of Toca 511 and Toca FC trial in high grade gliomas (NCT01470794) have been released. A safe dose has been established and as of the last report (August 25, 2017), several long-term survivors are still being followed (192).

The remaining clinical trials are listed in Table 2 and have not released any results to our knowledge.

**Table 2:** Clinical Trials targeting myeloid cells in combination with other anti-cancer therapies.

**Myeloid target**	**ClinicalTrials.gov identifier**	**Phase**	**Tumor types**	**Additional treatment**	**Results?**
CCR2	NCT03767582	1/2	Locally advanced pancreatic ductal adenocarcinoma (PDAC), Pancreatic ductal adenocarcinoma	Nivolumab + Stereotactic body radiation ± GVAX	–
	NCT03778879	1/2	Adenocarcinoma of the pancreas, Pancreas cancer	Stereotactic body radiation	–
CD40	NCT03597282	1	Metastatic melanoma	NEO-PV-01 (peptide vaccine) + Nivolumab (anti-PD-1)	–
Chemodepletion	NCT03302247	2	Non-small cell lung cancer stage IIIB	Nivolumab	–
COX-2	NCT03638297	2	Colorectal cancer	BAT1306 (anti-PD-1)	–
	NCT03864575	2	Metastatic cancer	Nivolumab	–
CSF-1R	NCT02452424	1/2a	Melanoma, Non-small cell lung cancer, Squamous cell carcinoma of the head and neck, Gastrointestinal stromal tumor (GIST), Ovarian cancer	Pembrolizumab (anti-PD-1)	–
	NCT02777710	1	Colorectal cancer, Pancreatic cancer, Metastatic cancer, Advanced cancer	Durvalumab (anti-PD-L1)	–
	NCT03238027	1	Solid tumor, Metastatic tumor, Locally advanced malignant neoplasm, Unresectable malignant neoplasm	Durvalumab	–
	NCT02880371	1/2	Advanced solid tumors	Pembrolizumab	–
	NCT02829723	1/2	Advanced solid tumors	PDR001 (anti-PD-1)	–
	NCT03336216	2	Advanced pancreatic cancer	Nivolumab + Chemotherapy	–
	NCT02526017	1a/1b	Advanced solid tumors, Including but not limited to lung cancer, Head and neck cancer, Pancreatic cancer, Ovarian cancer, Renal cell carcinoma, Malignant glioma	Nivolumab	Some preliminary efficacy observed along with macrophage depletion (183)
CSF-1R + CD40	NCT03502330	1	Advanced melanoma non-small cell lung cancer renal cell carcinoma	Nivolumab	–
CSF-1R + chemodepletion	NCT03697564	2	Pancreatic cancer stage IV	Nivolumab	–
CSF-1R + GM-CSF	NCT03153410	1	Pancreatic cancer	Pembrolizumab	–
GM-CSF	NCT00254592	2	Breast cancer	Bevacizumab OR Trastuzumab + chemotherapy	–
	NCT01498328	2	Glioblastoma, gliosarcoma	Rindopepimut (EGFRvIII peptide vaccine) + Bevacizumab	Treatment demonstrated improved ORR and PFS at 6 months compared to control (184)
GM-CSF vaccine	NCT01551745	2	Stage III, IV ovarian cancer	Bevacizumab	Study terminated due to no patients being analyzed for primary outcomes
	NCT01903330	2	Glioblastoma, Gliosarcoma	Bevacizumab + Cyclophosphamide (CY)	Increased survival in combination treatment over bevacizumab alone
GVAX	NCT01896869	2	Metastatic pancreatic adenocarcinoma	Ipilimumab (anti-CTLA-4)	–
	NCT02451982	1/2	Pancreatic cancer	Nivolumab ± Urelumab (anti-41BB)	–
	NCT03161379	2	Pancreatic cancer	CY + Nivolumab + Stereotactic body radiation	–
	NCT03190265	2	Pancreatic cancer	CY + Nivolumab + Ipilimumab + CRS-207 (listeria)	–
	NCT02243371	2	Pancreatic cancer	CY + CRS-207 + Nivolumab	–
	NCT03006302	2	Metastatic pancreatic adenocarcinoma	Epacadostat + Pembrolizumab + CRS-207	–
	NCT02648282	2	Pancreatic cancer	CY + Pembrolizumab + Stereotactic body radiation	–
	NCT01510288	1	Prostate cancer	Ipilimumab	Terminated due to company action
	NCT02981524	2	Metastatic colorectal cancer	CY + Pembrolizumab	Treatment is well-tolerated, results comparable to historical controls (186)
Pexa-Vec (GM-CSF)	NCT03294083	1	Renal cell carcinoma	REGN2810 (anti-PD-1)	
	NCT02562755	3	Hepatocellular carcinoma (HCC)	Sorafenib	
	NCT03206073	1	Refractory colorectal cancer	Durvalumab ± Tremelimumab (anti-CTLA-4)	Durvalumab safety established (193)
	NCT02977156	1	Metastatic/advanced solid tumors	Ipilimumab	
	NCT02630368	1/2	Solid tumors soft-tissue sarcoma breast cancer	CY	Safety established (194)
	NCT03071094	1/2a	Hepatocellular carcinoma (HCC)	Nivolumab	
PI3Kγ	NCT02637531	1	Advanced solid tumors non-small cell lung cancer melanoma squamous cell cancer of the head and neck triple negative breast cancer adrenocortical carcinoma mesothelioma high-circulating myeloid-derived suppressor cells	Nivolumab	Treatment is tolerable and results in some preliminary immunological activity, with reduced immunosuppression by macrophages (187)
Reparixin (CXCR1)	NCT02001974	1b	Breast cancer	Paclitaxel	–
STING	NCT03172936	1	Solid tumors and lymphomas	PDR001 (anti-PD-1)	–
	NCT03010176	1	Solid tumors and lymphomas	Pembrolizumab	Dose escalation ongoing, some signs of immunological activity (189)
	NCT02675439	1	Advanced/metastatic solid tumors or lymphomas	Ipilimumab	In STING agonist monotherapy arm, no dose-limiting toxicities yet, most patients dropped out due to disease progression or death, some immunological activity seen (190)
SX-682 (CXCR1/2)	NCT03161431	1	Melanoma	Pembrolizumab	–
Toca	NCT02576665	1	Colorectal cancer, triple negative breast cancer, Pancreatic cancer, non-small cell lung cancer, Head and neck cancer, Ovarian cancer, Lymphoma, sarcoma, Bladder cancer, Melanoma, IDH1 Mutated solid tumors, IDH1 Mutated or MGMT methylated recurrent HGG (Not Recruiting)	None	–
	NCT01985256	1	Glioblastoma multiforme, Anaplastic astrocytoma, Anaplastic oligodendroglioma, Anaplastic oligoastrocytoma	None	–
	NCT01156584	1	Glioblastoma multiforme, Anaplastic astrocytoma, Anaplastic oligodendroglioma, Anaplastic oligoastrocytoma	None	–
	NCT02598011	1	Newly diagnosed high grade glioma (HGG)	None	–
	NCT02414165	2/3	Glioblastoma multiforme, Anaplastic astrocytoma	None	–
	NCT01470794	1	Glioblastoma multiforme, Anaplastic astrocytoma, Anaplastic oligodendroglioma, Anaplastic oligoastrocytoma	None	Safe dose established, survival improved from external control, all responders remain alive as of August 25, 2017 (192, 195)
TVEC (GM-CSF)	NCT01161498	3	Squamous cell carcinoma head and neck cancer	Cisplatin	Terminated to permit redesign (196)
	NCT03597009	1b/2	Malignant pleural effusion stage IV metastatic cancer lung cancer	Nivolumab	
	NCT01740297	1b/2	Melanoma	Ipilimumab + TVEC	Approximate doubling of ORR in combination (191)
	NCT03802604	1	Breast cancer	Atezolizumab (anti-PD-L1)	
	NCT03069378	2	Sarcoma	Pembrolizumab	
	NCT02819843	2	Melanoma merkel cell carcinoma other solid tumors	Radiotherapy	
	NCT02978625	2	Non-melanoma skin cancer	Nivolumab	
	NCT03747744	1	Melanoma	Myeloid DCs	
	NCT03886311	2	Sarcoma	Nivolumab + Trabectedin	
	NCT02779855	1/2	Breast cancer	Chemotherapy	
	NCT02923778	2	Soft tissue sarcoma	Radiotherapy	
	NCT03256344	1	Metastatic triple negative breast cancer metastatic colorectal cancer	Atezolizumab	
	NCT02965716	2	Stage III-IV melanoma	Pembrolizumab	
	NCT03300544	1	Locally advanced or metastatic rectal cancer	Chemotherapy + Radiation therapy	
	NCT02626000	1	Carcinoma of the head and neck	Pembrolizumab	Combination safety established, showed some clinical effect (197)
	NCT03554044	1	Metastatic, unresectable, or Recurrent HER2- negative breast cancer	Chemotherapy OR Endocrine therapy	
	NCT02509507	1	Liver tumors	Pembrolizumab	
	NCT03088176	1	BRAF mutated advanced melanoma	BRAF/MEK inhibitors	

## Conclusion

A growing body of work has highlighted the importance of the myeloid compartment in glioma and other cancers. The composition of myeloid cells in the TME contributes to the success of immunotherapy as well as adjuvant treatments such as radiation and chemotherapy. Often, it is necessary to modulate the myeloid compartment to a phenotype that is pro-inflammatory to exert enhanced anti-tumor effects. Unfortunately, these effects are not often considered when designing new therapies. Additionally, as discussed above, the effect of certain myeloid modulators such as GM-CSF and tyrosine kinase inhibitors can change depending on the context or timing of treatment. To produce the desired treatment outcomes, it is necessary to thoroughly evaluate the therapeutic mechanism of myeloid targets. In this review, we have listed examples of combination therapies that have attempted to modulate the myeloid compartment in ways that improve the efficacy of the other treatments. These treatments introduce further challenges in ensuring that the separate treatments do not interfere, and instead synergize by addressing their respective deficits. Finally, we discussed the clinical trials attempting to target myeloid cells in combination with other therapies. Still a largely unanswered question regarding the myeloid compartment revolves around which patient populations and tumor types will effectively respond to myeloid-targeting therapy. While tumors with MDSC-enriched TMEs would presumably benefit from these strategies, other influencing factors have yet to be elucidated. Factors that may impact therapeutic efficacy may include levels of myeloid cells in the periphery that are eligible for recruitment, density of myeloid cells and their distance from the tumor core, the composition of immunomodulatory factors secreted by the TME, and the mutational landscape of the tumor itself. Although there is some promise of long-term survivors and responses from limited published trial results and as we eagerly await the results of various ongoing trials, it is important to continue to consider the above factors in the design of new combinations. We hope that with improved understanding of the complex interplay between various immune compartments in the TME and continued consideration of the role of myeloid cells in glioma and other tumor types, the efficacy of immune-based therapies will continue to improve with optimally designed combination treatment regimens.

## Author Contributions

Several authors were instrumental in making this manuscript. DR, AD, CJ, and ML contributed to the original conceptualization, writing, and editing of the manuscript. DR and AD contributed to the generation of visualizations associated with the manuscript.

### Conflict of Interest Statement

ML is a consultant for Tocagen, SQZ Technologies, Stryker, Baxter, and VBI. The remaining authors declare that the research was conducted in the absence of any commercial or financial relationships that could be construed as a potential conflict of interest.
